# Reduced Susceptibility to Sugar-Induced Metabolic Derangements and Impairments of Myocardial Redox Signaling in Mice Chronically Fed with D-Tagatose when Compared to Fructose

**DOI:** 10.1155/2018/5042428

**Published:** 2018-09-19

**Authors:** Debora Collotta, Laura Lucarini, Fausto Chiazza, Alessia Sofia Cento, Mariaconcetta Durante, Silvia Sgambellone, Jacopo Chini, Francesca Baratta, Manuela Aragno, Raffaella Mastrocola, Emanuela Masini, Massimo Collino

**Affiliations:** ^1^Department of Drug Science and Technology, University of Turin, Italy; ^2^Department of NEUROFARBA, University of Florence, Italy; ^3^Department of Clinical and Biological Sciences, University of Turin, Italy; ^4^Inalco RSM S.p.a, Research Center, Montale, Pistoia, Italy

## Abstract

**Background:**

D-tagatose is an isomer of fructose and is ~90% as sweet as sucrose with less caloric value. Nowadays, D-tagatose is used as a nutritive or low-calorie sweetener. Despite clinical findings suggesting that D-tagatose could be beneficial in subjects with type 2 diabetes, there are no experimental data comparing D-tagatose with fructose, in terms of metabolic derangements and related molecular mechanisms evoked by chronic exposure to these two monosaccharides.

**Materials and methods:**

C57Bl/6j mice were fed with a control diet plus water (CD), a control diet plus 30% fructose syrup (L-Fr), a 30% fructose solid diet plus water (S-Fr), a control diet plus 30% D-tagatose syrup (L-Tg), or a 30% D-tagatose solid diet plus water (S-Tg), during 24 weeks.

**Results:**

Both solid and liquid fructose feeding led to increased body weight, abnormal systemic glucose homeostasis, and an altered lipid profile. These effects were associated with vigorous increase in oxidative markers. None of these metabolic abnormalities were detected when mice were fed with both the solid and liquid D-tagatose diets, either at the systemic or at the local level. Interestingly, both fructose formulations led to significant Advanced Glycation End Products (AGEs) accumulation in mouse hearts, as well as a robust increase in both myocardial AGE receptor (RAGE) expression and NF-*κ*B activation. In contrast, no toxicological effects were shown in hearts of mice chronically exposed to liquid or solid D-tagatose.

**Conclusion:**

Our results clearly suggest that chronic overconsumption of D-tagatose in both formulations, liquid or solid, does not exert the same deleterious metabolic derangements evoked by fructose administration, due to differences in carbohydrate interference with selective proinflammatory and oxidative stress cascades.

## 1. Introduction

Excess of sugar consumption has been linked epidemiologically with the development of cardiometabolic diseases [1–4]. Among the most widely added sugars used, fructose has been a source of great concern, according to several studies showing that high-fructose intake is a main driver of a whole range of metabolic and cardiovascular alterations in animal and human models [5]. High dietary fructose intake promotes the development of pathological characteristics leading to obesity, insulin resistance, and type 2 diabetes [6, 7]. Epidemiologic evidence has been confirmed by several experimental data, showing that consumption of high fructose in beverages causes metabolic syndrome phenotype in rodents [8]. However, the mechanism through which excessive fructose intake causes dysmetabolic effects is not completely known. Fructose is absorbed in the small intestine by specific facilitative transporters expressed in both the apical and basolateral membrane of enterocytes. After absorption, fructose is primarily metabolized in the liver, which takes up at least 50% of the initial fructose flux. We have contributed to deepen the molecular mechanisms leading to the peculiar lipid deposition induced by excessive fructose consumption in ectopic tissues, such as the liver and skeletal muscle. Specifically, we showed that the high chemical reactivity of fructose substantially contributes to the massive formation of intracellular Advanced Glycation End Products (AGEs), thus evoking marked cellular alterations and organ dysfunction [9–14]. More recently, we documented that consumption of different fructose formulations, liquid or solid, evokes different impacts on gut microbiota and integrity, thus differently affecting liver homeostasis [15]. In order to counteract the growing burden of sugar-related chronic diseases, innovative strategies, aimed to reduce caloric intake and to replace conventional sugars with new low caloric sweeteners, have garnered increasing attention. Among the available alternative sugars, one of the most promising is the D-tagatose, a peculiar sugar that is structurally similar to D-fructose and has high palatability and a good sweetening property and is low in calories [16, 17]. This natural ketohexose is proved to be a potential replacement to sucrose-like high-calorie bulk sweeteners as food additives, thanks to its poor absorption and metabolization within the human body [18]. Unlike fructose that has high glycation capacity and promotes lipogenesis, D-tagatose has a lower glycation index [19]. In human subjects, D-tagatose lowered postprandial blood glucose and insulin response allegedly through inhibiting intestinal disaccharides as like as glucose transport. In addition, the results of a phase 3 clinical trial illustrated the potential for D-tagatose, which effectively lowered HbA1c levels in type 2 diabetic patients compared to placebo [20–22]. Unfortunately, the molecular mechanisms underlying health effects of the low caloric sugar D-tagatose have hardly been investigated. To our knowledge, there is only an *in vivo* study documenting the effects of D-tagatose on obesity, hyperglycemia, and hypercholesterolemia in mice [23]. So far, no experimental data are available comparing D-tagatose with fructose, in terms of metabolic derangements and related molecular mechanisms evoked by chronic exposure to high levels of these two different carbohydrate sources. Thus, the aim of this study was to determine if and how D-tagatose alters lipid and sugar metabolism in comparison to the results obtained with fructose, well known for its glycative and lipogenic potential. Furthermore, since we recently demonstrated that chronic feeding with fructose contributes to evoke a maladaptive response in cardiac tissue [24], we accordingly investigated the potential different impacts of the two sugars on the heart, focusing mainly on selective signaling pathways involved in the cardiometabolic disease pathogenesis.

## 2. Materials and Methods

### 2.1. Animal Model and Procedures

Four-week-old male C57Bl/6j mice (Charles River Laboratories, Calco, Italy, *n* = 30) were cared in conformity with the European Council directives (No. 2010/63/EU) and with the Principles of Laboratory Animal Care (NIH No. 85-23, revised 2011). The scientific project was approved by the Ethical Committee of Turin University and by the Italian Ministry of Health (Authorization No. 1189/2016/PR). Mice, housed in a temperature-controlled environment with a 12 h light/dark cycle, were randomly allocated into the following dietary regimens: a group fed a control diet and drinking tap water (CD group, *n* = 6), a group fed a control diet and drinking a 30% fructose syrup (L-Fr group, *n* = 6), a group fed with 30% fructose solid diet and drinking tap water (S-Fr group, *n* = 6), a group fed a control diet and drinking a 30% D-tagatose syrup (L-Tg group, *n* = 6), and a group fed with 30% D-tagatose solid diet and drinking tap water (S-Tg group, *n* = 6) during 24 weeks. All groups received drink and food *ad libitum*. All along the experimental protocol, body weight, glycemia, and food intake were strictly monitored. One day before euthanasia, urine of mice was collected for analysis by placing animals in metabolic cages for 18 h.

Mice were anesthetized and killed by operating cardiac exsanguination, so the blood was collected and the heart was rapidly removed, frozen in liquid N_2_, and stored at −80°C in order to carry out protein analysis.

### 2.2. Plasma and Urine Biochemical Analyses

A plasma lipid profile (triglycerides [TGs], high-density lipoprotein [HDL], and low-density lipoprotein [LDL]) was determined by standard enzymatic procedures using a reagent kit (Hospitex Diagnostics, Florence, Italy). The concentration of plasma creatinine was assessed by using commercial kits (Arbor Assays, Ann Arbor, MI, USA).

HbA1C and proteinuria were measured in plasma and urine, respectively, by using standard analysis kits. The plasma leptin level and inflammatory profile (TNF-*α*, IL-1*β*, and IL-6) were determined, as previously described [25] using enzyme-linked immunosorbent assay (ELISA) kits.

### 2.3. Evaluation of Myocardial Oxidative Stress

#### 2.3.1. Determination of Malondialdehyde (MDA)

MDA was determined by measurement of the chromogen obtained from the reaction of MDA with 2-thiobarbituric acid. Briefly, heart tissues were homogenized with 1 ml of 50 mM Tris-HCl buffer containing 180 mM KCl and 10 mM EDTA, final pH 7.4. Then, 0.5 ml of 2-thiobarbituric acid (1% *w*/*v*) in 50 mM NaOH and 0.5 ml of HCl (25% *w*/*v* in water) were added to 0.5 ml of sample. The mixture was placed in test tubes, sealed with screw caps, and heated in boiling water for 10 min. After cooling, the chromogen was extracted in 3 ml of 1-butanol, and the organic phase was separated by centrifugation at 2000*g* for 10 min. The absorbance of the organic phase was read spectrophotometrically at a 532 nm wavelength. The values are expressed as nanomoles of thiobarbituric acid-reactive substances (MDA equivalents) per milligram of protein, using a standard curve of 1,1,3,3-tetramethoxypropane.

#### 2.3.2. Evaluation of Catalase (CAT)

CAT activity was measured using the Calbiochem® Catalase Assay Kit (Merck Millipore) following the instructions provided by the manufacturer.

#### 2.3.3. Determination of 8-Hydroxy-2′-deoxyguanosine (8-OHdG)

DNA isolation from cardiac tissue homogenates was performed according to Collino et al. [25, 26]. Samples of DNA extract were used for 8-hydroxy-2′-deoxyguanosine (8-OHdG) determination using an ELISA kit (JalCA, Shizuoka, Japan), following the instructions provided by the manufacturer. The absorbance of the chromogenic product was measured at 450 nm and expressed as ng/mg of DNA. The results were calculated from a standard curve based on 8-OHdG solution. The values are expressed as ng 8-OHdG/*μ*g total DNA.

### 2.4. Protein Extraction and Western Blot

Cytosolic and nuclear extracts from hearts were prepared as previously described [27]. Succinctly, hearts were homogenized at 10% (wt/vol) with a Potter Elvehjem homogenizer (Wheaton, Millville, NJ) using a homogenization buffer containing 20 mM HEPES (pH 7.9), 1 mM MgCl_2_, 0.5 mM EDTA, 1% Nonidet P-40, 1 mM EGTA, 1 mM DTT, 0.5 mM PMSF, and 1 *μ*l/ml of PIC. Homogenates were centrifuged at 1300*g* for 5 min at 4°C. Supernatants were removed and centrifuged at 16,000*g* at 4°C for 40 min to obtain supernatant containing the cytosolic fraction. The pelleted nuclei were resuspended in extraction buffer (1/3 volume of the homogenation buffer) containing 20 mM HEPES (pH 7.9), 1.5 mM MgCl2, 300 mM NaCl, 0.2 mM EDTA, 20% glycerol, 1 mM EGTA, 1 mM DTT, 0.5 mM PMSF, and 1 *μ*l/ml of PIC and incubated in ice for 30 min, followed by centrifugation at 16,000*g* for 20 min at 4°C. The resulting supernatants containing nuclear proteins were carefully removed. Protein content was determined by the BCA assay, and extracts were stored at −80°C until use. Semiquantitative immunoblot analyses of the phosphorylation of IKK*α*/*β* and I*κ*B*α*, nuclear translocation of p65, expression of COX-2 and RAGE, and CML- and CEL-glycated proteins were carried out in mouse heart tissue extracts. About 60 *μ*g of proteins were separated by SDS-PAGE and electrotransferred on a PVDF membrane. After blocking (1 h in 5% dry milk solution), membranes were incubated with primary antibodies (rabbit anti-p65 NF-*κ*B [1 : 1000], rabbit anti-IKK*α*/*β* [1 : 1000], rabbit anti-Ser^176/180^ IKK*α*/*β* [1 : 5000], mouse anti-I*κ*B*α* [1 : 1000], mouse anti-Ser^32/36^ I*κ*B*α* [1: 1000], rabbit anti-COX-2 [1 : 1000], goat anti-RAGE, [1 : 1000], mouse anti-CML [1 : 500], and mouse anti-CEL [1 : 100]) followed by incubation with appropriated HRP-conjugated secondary antibodies. To ascertain that membranes were loaded with equal amounts of cytosolic or nuclear proteins, they were also incubated with antibody against tubulin or histone-H3, respectively. Proteins were detected with an enhanced chemiluminescent (ECL) detection system and quantified by densitometry using an analytic software (Quantity-One, Bio-Rad, Hercules, CA, USA). Results were normalized with respect to densitometric value of the protein used as loading control.

### 2.5. PgE_2_ Measurement

Tissue fragments were homogenized at 0–4°C in the presence of 10 *μ*mol/liter indomethacin so as to prevent PG production during the procedure, and then they were centrifuged at 600*g*. Five hundred *μ*l of tissue homogenates were used for PgE_2_ determination using a competitive enzyme immunoassay kit (Cayman Chemical Co., Ann Arbor, MI).

### 2.6. Statistics

All values are presented as mean ± SEM for no. observations. We analyzed data using the Prism software package (GraphPad Software, San Diego, CA, USA). Comparisons among groups were performed using one-way ANOVA with Bonferroni's multiple comparison post hoc test. Differences between groups were considered statistically significant at *p* values below 0.05.

## 3. Results

### 3.1. Effects of Fructose- or D-Tagatose-Enriched Diets on Metabolic Parameters

As shown in Table 1, chronic exposure to Fr diet evoked a two-fold increase in body weight gain, when compared to CD at 24 weeks of dietary manipulation. In contrast, body weight gain recorded in mice exposed to D-tagatose was similar to that recorded in control mice.

After 24 weeks of dietary manipulation, L-Fr and S-Fr mice showed a significant worsening in glycemic profile (Table 1), with an increase in plasma fasting glucose and in the percentage of HbA1c, if compared to CD- or D-tagatose-fed mice. Similarly, L-Fr and S-Fr diets led to an increase in plasma leptin concentration and in the levels of blood triglycerides and LDL; paralleled by a significant decrease in HDL levels. Leptin and lipid profile were not affected by L-Tg or S-Tg diets.

Fructose (both liquid and solid formulations), but not D-tagatose, reduced kidney function, leading to a significant increase in blood serum creatinine and proteinuria, when compared to control mice.

### 3.2. Effects of Fructose- or D-Tagatose-Enriched Diets on Markers of Systemic Inflammation

**Figure 1:**
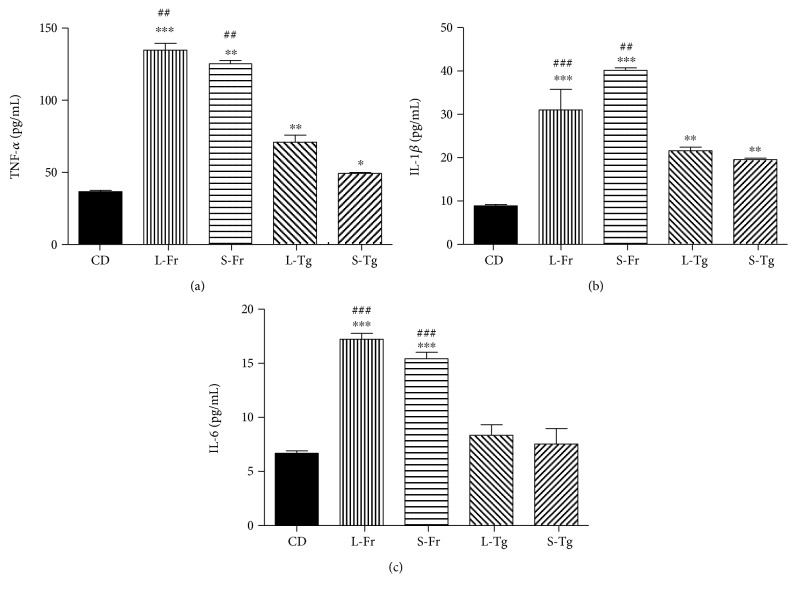
Effects of chronic sugar exposure on systemic inflammatory profile. Plasma levels of TNF-*α* (a), IL-1*β* (b), and IL-6 (c) were measured in mice fed for 24 weeks with a control diet or with a diet containing liquid or solid supplementation of fructose/D-tagatose. Values are represented as means ± S.E.M. of 6 animals per group. ^∗^*p* < 0.05, ^∗∗^*p* < 0.01, and ^∗∗∗^*p* < 0.001, vs. CD. ## and ### denote, respectively, *p* < 0.01 and *p* < 0.001 vs. corresponding Tg formulation.

Chronic supplementation with either fructose or D-tagatose, both in liquid and solid form, led to a significant increase in plasma levels of TNF-*α* and IL-1*β* when compared to CD (Figure 1). However, the increase in TNF-*α* and IL-1*β* evoked by D-tagatose feeding reached values that were approximately halved if compared to the levels obtained in the corresponding fructose diet group. Interestingly, no effects of D-tagatose on IL-6 plasma levels were recorded, whereas fructose chronic exposure induced a three-fold increase in this systemic inflammatory marker.

### 3.3. Effects of Fructose- or D-Tagatose-Enriched Diets on Myocardial Markers of Oxidative Stress

Sugar-sweetened diet can intensely contribute to the development of heart disease [28, 29] mainly increasing oxidative stress [30].

**Figure 2:**
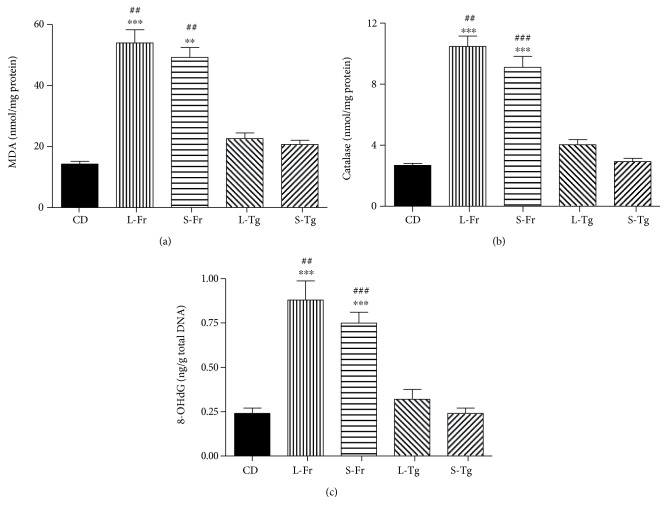
Effects of sugars feeding on myocardial markers of oxidative stress. Malondialdehyde (MDA; a), catalase (b), and 8-hydroxy-2′-deoxyguanosine (8-OHdG; c) were measured in heart homogenates of mice fed for 24 weeks with a control diet or with a diet containing liquid or solid supplementation of fructose/D-tagatose. Values are represented as means ± S.E.M. of 6 animals per group. ∗∗ and ∗∗∗ denote, respectively, *p* < 0.01 and *p* < 0.001 vs. CD. ## and ### denote, respectively, *p* < 0.01 and *p* < 0.001 vs. corresponding Tg formulation.

When measured in the hearts of mice fed a diet enriched in liquid or solid fructose, the oxidative stress markers (MDA, catalase, and 8-OHdG) were tripled compared to those of the CD for both diet regimens, without significant differences between the liquid or solid form (Figure 2). None of these oxidative derangements was observed in the heart tissue homogenates of D-tagatose-fed mice.

### 3.4. Effects of Fructose- or D-Tagatose-Enriched Diets on Myocardial Activation of Inflammatory Pathways

Since the results so far obtained did not show any significant difference between the liquid and the solid administration regime, both at the systemic or myocardial level, the evaluation of the molecular mechanisms underlying the different effects of fructose or D-tagatose administration on the heart was performed in the two liquid groups only.

**Figure 3:**
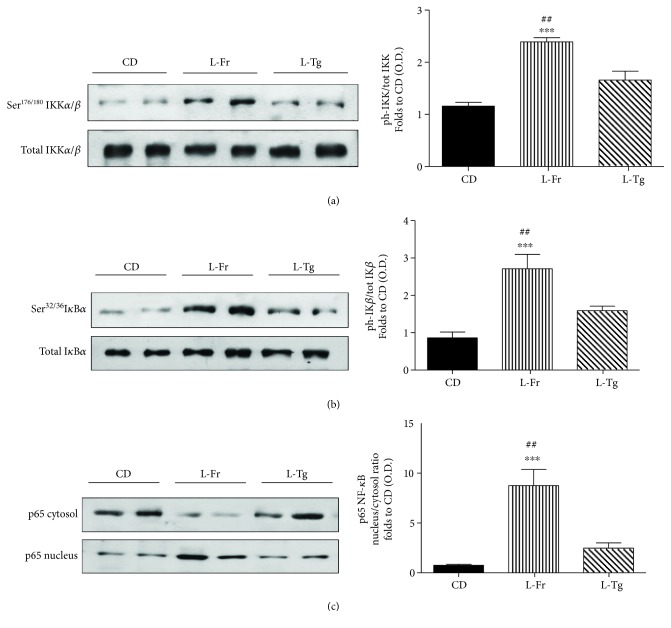
Effects of liquid sugars feeding on myocardial activation of the NF-*κ*B pathway. Representative Western blotting and relative densitometric analysis of the activation of the NF-*κ*B pathway measured as phosphorylation of IKK*α*/*β* at Ser^176/180^ (a), phosphorylation of I*κ*B*α* at Ser^32/36^ (b), and nuclear translocation of p65 subunit of NF-*κ*B (c) in the heart of mice fed for 24 weeks with a control diet or with a diet containing liquid supplementation of fructose/D-tagatose. Protein expression was measured as relative optical density (OD), corrected for the corresponding tubulin or histone H3 contents and normalized using the related CD band. Values are represented as means ± S.E.M. of 6 animals per group. ∗∗∗ denotes *p* < 0.001 vs. CD. ## denotes *p* < 0.01 vs. L-Tg diet.

We investigated the effects of fructose or D-tagatose supplementation on the signaling pathways involved in the activation of NF-*κ*B, as this transcriptional factor plays a pivotal role in diet-induced inflammation. When compared to CD-fed mice, we observed a significant increase in the phosphorylation of Ser^176/180^ on IKK*α*/*β* (Figures 3(a)) and Ser^32/36^ on I*κ*B*α* (Figures 3(b)) and the nuclear translocation of the p65 NF-*κ*B subunit (Figures 3(c)) in the heart of fructose-fed mice. D-tagatose supplementation did not significantly affect the phosphorylation of Ser^176/180^ on IKK*α*/*β* and Ser^32/36^ on I*κ*B*α* nor the subsequent translocation of the p65 NF-*κ*B subunit from the cytosol to the nucleus (Figure 3).

**Figure 4:**
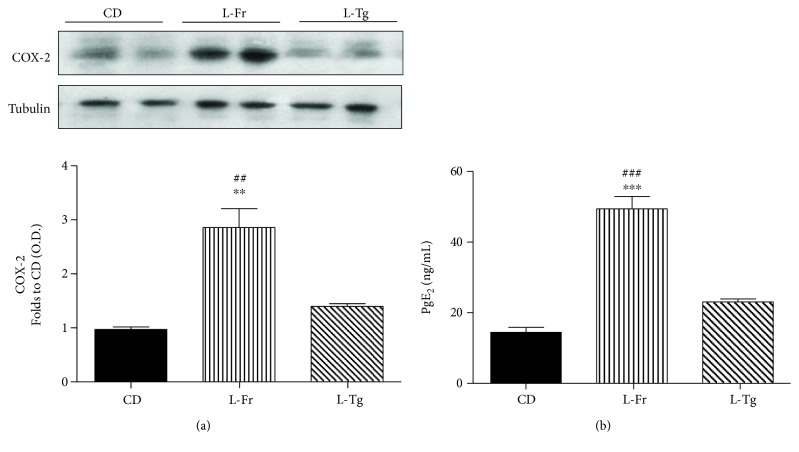
Effects of liquid sugars feeding on myocardial expression and activation of COX-2. Representative Western blotting and relative densitometric analysis of the expression of COX-2 (a) and concentration of PgE2 (b) in the heart of mice fed for 24 weeks with a control diet or with a diet containing liquid supplementation of fructose/D-tagatose. Protein expression was measured as relative optical density (OD), corrected for the corresponding tubulin contents and normalized using the related CD band. Values are represented as means ± S.E.M. of 6 animals per group. ∗∗ and ∗∗∗ denote, respectively, *p* < 0.01 and *p* < 0.001 vs. CD. ## and ### denote, respectively, *p* < 0.01 and *p* < 0.001 vs. L-Tg diet.

The local excessive inflammatory response within the heart of fructose-fed mice was further confirmed by the detection of increased myocardial expression and activation of the COX-2 enzyme isoform. In fact, when compared to CD-fed mice, a significant increase in the expression of COX-2 and release of PgE_2_ in the heart of fructose chronically fed mice was observed. D-tagatose supplementation did not significantly affect COX-2 expression and activity (Figure 4).

### 3.5. Effects of Fructose- or D-Tagatose-Enriched Diets on Myocardial Activation of the AGE/RAGE Cascade

**Figure 5:**
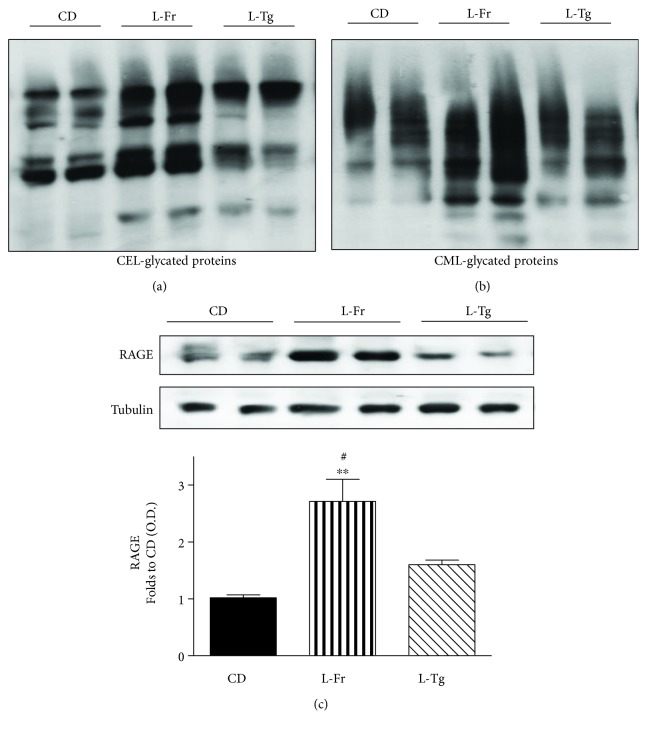
Effects of liquid sugars feeding on myocardial accumulation of AGE and expression of RAGE. Representative Western blotting and relative densitometric analysis of CEL- and CML-glycated proteins (a and b, respectively) and RAGE expression (c) in the heart of mice fed for 24 weeks with a control diet or with a diet containing liquid supplementation of fructose/D-tagatose. Protein expression was measured as relative optical density (OD), corrected for the corresponding tubulin contents and normalized using the related CD band. Values are represented as means ± S.E.M. of 6 animals per group. ∗∗ denotes *p* < 0.01 vs. CD. # denotes *p* < 0.05 vs. L-Tg diet.

Advanced Glycation End Products (AGEs) are toxic compounds deriving from nonenzymatic glycation reactions of reducing sugars with proteins, which then result in becoming structurally and functionally compromised. AGEs are known to contribute to the development and/or progression of cardiovascular diseases, mainly through induction of oxidative stress and inflammation. Here we measured local levels of the most known AGEs carboxymethyllysine/carboxyethyllysine- (CML/CEL-) protein adducts, showing a massive overproduction in the group of mice fed fructose liquid formulation (Figures 5(a) and 5(b)). As expected, the fructose effects on myocardial accumulation of AGEs are parallel with an almost three-fold increase in RAGE expression in the L-Fr group when compared to CD mice (Figure 5(c)). Interestingly, Western blot analysis demonstrated that D-tagatose feeding was associated with a significantly lower AGE accumulation and RAGE hyperexpression when compared to those recorded in the L-Fr group.

## 4. Discussion

Our results confirm and further extend previous studies demonstrating the damaging effects associated with chronic exposure to high-fructose intake, leading to impairments of systemic glucose and lipid profiles as well as renal function. Most notably, we documented that the metabolic abnormalities caused by exposure to an unhealthy diet containing high concentrations of fructose were not recorded when fructose was replaced by D-tagatose, thus demonstrating that the proposed change in nutrient composition favorably affected metabolic homeostasis. Accordingly, D-tagatose had no effect on body weight, whereas a diet containing an equivalent amount of fructose resulted in significant body weight gain at the end of the dietary manipulation. These findings are in keeping with a previously published paper showing that, in comparison to sucrose, a diet enriched in D-tagatose as a carbohydrate source did not promote obesity, hyperglycemia, or adipocyte hypertrophy and resulted in a lesser extent of hypercholesterolemia and atherosclerosis, when tested in low-density lipoprotein receptor-deficient (LDLr^−/−^) mice [23]. Our study was designed to perform also a comparative investigation on the toxicological impact of different formulations (liquid versus solid forms) of the same dietary component. Our data show no differences for systemic metabolic and inflammatory parameters according to the sugar format. Interestingly, the systemic inflammatory profile recorded in the blood of D-tagatose-fed mice was far less than that observed in fructose-fed mice. The excessive systemic inflammation of fructose-fed mice may have significantly contributed to glucose and lipid dysregulation, with the role of a low-grade, chronic inflammatory response, known as metaflammation, in promoting metabolic diseases being well described [31]. In contrast, the limited inflammatory profile in D-tagatose-fed mice is suggestive, at least in part, of a reduced secretion of inflammatory mediators by obese visceral fat, thanks to the D-tagatose ability to counteract mice weight gain. Experimental evidence supports a role for components of the diet, including sugar-sweetened beverages, in the development of heart disease [28, 29]. As confirmed by several epidemiological studies in diabetic patients, cardiovascular complications represent the principal cause of morbidity and mortality. The mechanisms of diabetic cardiomyopathy are multifaceted, involving increased oxidative/nitrosative stress, accumulation of AGEs, activation of various proinflammatory, and cell death signaling pathways [30]. Myocardial inflammation has been reported as one of the main driving forces in the pathogenesis of cardiac dysfunction, and notably, the linkage of metabolic cardiomyopathy with inflammation has incited research into new effective treatments for diastolic dysfunction of obesity and insulin resistance [32]. We have recently contributed to demonstrate a correlation between increased activity of selective inflammatory pathways, due to chronic high-fructose feeding, and enhanced susceptibility to a myocardial ischemic insult [24]. Interestingly, the circulating levels of the soluble receptor of AGEs (sRAGE) have been suggested to be a valuable predictor of cardiovascular diseases, being sRAGE concentrations elevated after myocardial infarction [33] and associated with poor in-hospital prognosis [34]. In keeping with these previous findings, we report here that fructose feeding resulted in overexpression of both AGEs and the chief AGE receptor RAGE in the myocardial tissue. Most notably, we offered, for the first time, the experimental evidence of the lower chemical reactivity of D-tagatose when compared to its epimer fructose, resulting in a dramatic decrease in the *in vivo* formation of AGEs and expression of RAGE. Thus, we can state that slight differences in the position of the hydroxyl group at carbon atom 4 may result in significant differences in sugar reactivity and sugar metabolism. Recent findings show that fructose produces 10 times more AGEs than glucose because the anomerization equilibrium for fructose is shifted more to the reactive, open chain form of the sugar [35]. Even though our study did not deepen the chemical characterization and quantification of the specific structures of sugar-induced protein glycation products, it is, to our knowledge, the first experimental comparative evidence of mechanistic differences associated with chronic exposure to two ketohexose stereoisomeric sugars. Oxidative stress is a key factor in the pathophysiology of diet-related cardiovascular complications [36, 37]. Very recently, RAGE activation, following binding with AGEs, has been shown to mediate oxidative stress in cardiomyocytes [38]. Here we confirmed that mice exposed to fructose, in both the liquid and solid formulations, exhibited a massive increase in myocardial oxidative stress, as suggested by markers of peroxidation of cell membrane lipids and radical-induced DNA damage as well as activity of antioxidant enzymes. The lack of any effects on the measured markers of oxidative stress by both solid and liquid formulation of D-tagatose further corroborate the evidence of reduced activation of the AGE/RAGE-dependent pathways. The activation of the AGE/RAGE signaling is known to be associated not only with increased oxidative stress but also with a proinflammatory state, due mainly to the activation and nuclear translocation of the NF-*κ*B transcription factor [39]. As recently documented in an elegant *in vivo* study [40], ingestion of AGE-enriched food resulted in a rapid activation of NF-*κ*B in the heart, via a direct interaction with RAGE, reaching the highest myocardial NF-*κ*B activity 12 h after AGE feeding. As reported here, fructose, but not D-tagatose, feeding evoked a massive activation of the IKK complex and the related degradation of I*κ*B*α*, thereby liberating NF-*κ*B, resulting in NF-*κ*B translocation to the nucleus. Long-term activation of the proinflammatory NF-*κ*B may account for the increased expression and activity of COX-2 recorded in the homogenates of hearts from fructose-fed mice. The role of COX-2 in heart function and pathology has been widely explored in the last few years, mainly due to the finding that specific COX-2 inhibitors significantly increased the risk of heart disease in chronic users [41]. Both elevated expression of COX-2 in cardiomyocytes and local concentrations of prostaglandins have been associated with human heart disease, particularly heart failure [42, 43]. Of course, such studies are quite inconclusive, as the recorded increases could be secondary effects due to the heart failure, as opposed to an ongoing mechanism of pathology. However, they overall suggest that impairments in COX-2 expression and activity may be associated with the development of heart diseases. Thus, the experimental evidence of nonsignificant modulation of both markers of oxidative stress and excessive inflammatory response in the heart of mice chronically fed D-tagatose is suggestive of reduced potential cardiac toxicity when D-tagatose is used as a sugar substitute. The experimental model proposed here allows us to perform a comparative study, in a strictly controlled environment, on the intrinsic capacity of two fructose epimers to affect an early marker of myocardial injury and dysfunction. However, we are aware of some limitations of the present study, including the lack of pharmacokinetics, hemodynamic and functional data, and the impossibility to dissect between the redox and inflammatory effects related to sugar exposure. Our study does not allow identifying the specific cell types involved in sugar-mediated responses. Cardiomyocytes are the most prominent cell type in the heart, and loss of contractile tissue is the most important consequence of a myocardial dysfunction. However, dysregulation of cardiac fibroblast function may also contribute to adverse cardiac remodeling and eventually heart failure, and AGEs have been demonstrated to induce cardiac fibroblast proliferation by interfering with the AGE/RAGE pathway [44]. Besides, as D-tagatose is partially fermented in the large intestine [45, 46], we cannot rule out that differences in absorption of the two fructose epimers may affect, at least in part, their different toxicological profiles. Thus, future *ad hoc* studies are required to clarify these aspects.

In conclusion, to the best of our knowledge, this is the first study that analyzes from a comparative point of view the consequences of chronic oral administration of fructose or its naturally occurring epimer, D-tagatose, in a nongenetic model of diet-induced metabolic derangements. Our data demonstrate significant qualitative and quantitative sugar-dependent differences in early markers of organ injury, due to selective interference of fructose with the AGE/RAGE cascade, not recorded when mice were fed with D-tagatose. Thanks to its very low caloric value and organoleptic similarities with fructose, the rare sugar D-tagatose may represent a safer paradigm of sweeteners with limited toxicological impact on obesity and associated metabolic disorders. Further preclinical and clinical studies are needed to better explore this possibility and to investigate/ensure the safety of this innovative approach.

## Figures and Tables

**Table 1:** Effects of fructose and tagatose on systemic parameters at 24 weeks of dietary manipulation.

	CD (*n* = 6)	L-Fr (*n* = 6)	S-Fr (*n* = 6)	L-Tg (*n* = 6)	S-Tg (*n* = 6)
Glucose (nmol/l)	11.3 ± 0.7	15.1 ± 0.4^∗∗^^§§^	14.6 ± 0.6^∗∗^^§§^	11.0 ± 0.2	10.9 ± 0.9
HbA1c (%)	2.8 ± 0.1	5.5 ± 0.1^∗∗^^§^	4.8 ± 0.2^∗∗^^§^	3.13 ± 0.4	3.0 ± 0.3
Leptin (*μ*g/ml)	2.4 ± 0.1	27.3 ± 0.7^∗∗^^§^	17.7 ± 1.2^∗∗^^§^	3.9 ± 0.8	5.1 ± 0.3
Triglycerides (mg/dl)	39.6 ± 3.9	64.8 ± 2.6^∗∗^^§§^	63.7 ± 4.6^∗∗^^§§^	38.7 ± 2.5	36.4 ± 3.6
HDL (mg/dl)	53.2 ± 5.6	34.7 ± 5.2^∗∗^^§§^	35.6 ± 5.0^∗∗^^§§^	44.7 ± 6.9	46.6 ± 3.4
LDL (mg/dl)	23.6 ± 2.4	36.5 ± 3.1^∗∗^^§§^	34.2 ± 4.1^∗∗^^§§^	29.6 ± 3.4	26.5 ± 5.2
Creatinine (mg/dl)	0.45 ± 0.06	1.15 ± 0.09^∗∗^^§§^	0.90 ± 0.08^∗∗^	0.68 ± 0.09	0.68 ± 0.07
Proteinuria (mg/24 hours)	6.13 ± 0.24	17.4 ± 0.33^∗∗^^§§^	14.63 ± 0.54^∗∗^^§§^	6.45 ± 0.42	6.60 ± 0.27
Body weight gain (g)	3.52 ± 0.26	6.00 ± 0.26^∗∗^^§§^	6.32 ± 0.50^∗∗^^§§^	2.64 ± 0.43	2.38 ± 0.27

Data are means ± S.E.M. ^∗∗^*p* < 0.01 vs. control diet ^§^*p* < 0.05 vs. tagatose; ^§§^*p* < 0.01 vs. tagatose.

## Data Availability

The data used to support the findings of this study are available from the corresponding author upon request.
